# Editorial: Emerging talents in human and medical genomics

**DOI:** 10.3389/fgene.2024.1497486

**Published:** 2024-09-30

**Authors:** Vanessa Meyer, Stephen J. Bush, Angela Botes, Mandeep Kaur

**Affiliations:** ^1^ School of Molecular and Cell Biology, University of the Witwatersrand, Johannesburg, South Africa; ^2^ School of Automation Science and Engineering, Xi’an Jiaotong University, Xi’an, Shaanxi, China

**Keywords:** emerging researchers, human genomics, medical genomics, maternal health, hearing loss, genomics applications

The field of human genomics has progressed extensively since the publication of the first draft of the human genome in 2001. A simple search in PubMed using the keywords “Human Genomics” produced a list of 5 521 articles published between the years 1990–2000, 15 740 in the first decade of the 21st century (2001–2010), 57 346 in the years 2011–2020, and from 2021 until 24th August 2024, 32 856 articles have been published. Genomics has evidently found its application in a variety of research areas, diversifying our knowledge and understanding of the biological mechanisms of health and disease. This broad topic provided a platform specifically to early-career researchers (the first or co-first authors were all either enrolled on under- or postgraduate programmes) to publish their work in the fields of human and medical genomics and enable them to experience the collaborative aspects of the publishing process and workflows. The topic highlights studies that have made innovative use of genomics technologies to address issues related to maternal health, surgical treatment, and hearing loss.

Lin et al. characterized the extrachromosomal circular DNA (eccDNA) from placental trophoblasts in placental and maternal plasma in a small number of samples to provide a proof-of-concept to use eccDNA as a biomarker for foetal growth restriction (FGR), a perinatal complication in foetuses. The authors performed eccDNA sequencing on a small number of samples and the analysis revealed the enrichment of both multi-chromosomal-fragment and single-fragment eccDNA in placenta, while only single-fragment eccDNA was identified in maternal plasma. The comparison of data from plasma between FGR and control groups led to the identification of abundant genes such as *IGF2* and *ZNF445* in the FGR group (linked to epigenetic regulation of gene expression and genomic imprinting), whereas *ARG1*, *CCND1*, *CYP1A1*, *EZR*, *GPR68*, *MYO1C*, and *XPO4* (linked to response to vitamins, response to nutrient levels, and positive regulation of protein transport) were found to be abundant genes in the control group. Additionally, placental eccDNA–associated genes, those which were associated with immunity-related gene ontology terms, showed differential abundance between the FGR and control groups. This integrated data analysis from both placental and maternal plasma, combined with genomic annotation, offered insights into the potential of using eccDNA as biomarker of FGR and opens doors for using maternal plasma eccDNA signatures for future investigations into pregnancy-related diseases and complications.

Qi et al. sheds light on a critical intersection of virology and reproductive health. This research is particularly timely given the ongoing global impact of COVID-19 and its far-reaching implications. The study examines the expression of SARS-CoV-2-associated molecules in the endometrium, revealing significant insights into how these factors vary throughout the menstrual cycle. One of the most compelling aspects of this study is its focus on women with recurrent pregnancy loss (RPL) and recurrent implantation failure (RIF). The significant reduction in TMPRSS2 (transmembrane protease serine 2) expression in the RIF group compared to the controls underscores the need for further investigation into how SARS-CoV-2 might exacerbate reproductive challenges. Similarly, in women with RPL, the peak expression of the host cell receptor basigin (BSG) during the window of implantation suggests a potential period of heightened vulnerability to SARS-CoV-2. Despite the limited sample numbers, this study is a crucial step toward understanding the interplay between viral infections and reproductive health and helps address the unique challenges faced by women with reproductive health issues.

Fernández-Boyano et al. innovatively applied bioinformatics and artificial intelligence to design a model (eoPred) that can be used to identify early-onset preeclampsia from DNA methylation data and predict placental phenotype regardless of cell composition. Preeclampsia is a life-threatening condition and the leading cause of maternal and infant illness and death in the US. The package, which is available at https://www.bioconductor.org/packages/release/bioc/html/planet.html, is expected to significantly enhance current approaches at identifying placental insufficiency and be useful in larger, more complex EWAS studies as well.

Turning from its use in maternal health, Kueng et al. shifts our focus to the practical use of genomics as a non-invasive tool to monitor allograft injury after solid organ transplants. The authors compared three methods currently used to quantify donor derived cell-free DNA (dd-cfDNA) and assessed the strengths and limitations of each. While the relative quantification of dd-cfDNA yielded comparable results between techniques, the absolute quantification methods showed more variability. The authors further present a custom high-throughput sequencing panel of 121 common polymorphisms linked to unique molecular identifiers that can be used to effectively quantify the percentage of dd-cfDNA in plasma and urine samples. Advances in these types of technologies and approaches are sure to improve the patient outcomes after major organ transplants through monitoring and early detection of allograft injury.

Finally, Imizcoz et al. devised a gene panel for the diagnostic sequencing of congenital hearing loss, globally the most prevalent of sensory disorders and which, in Europe, affects 1-2 newborns per thousand. Genetic diagnoses of hearing loss, in pinpointing pathogenic variants, can provide valuable information regarding prognosis and aetiology, with early diagnosis imperative for early intervention. To that end, the authors designed a panel encompassing the coding regions of 179 genes clinically related to a known type of hereditary hearing loss and assessed its utility on 155 patients with hearing loss for whom a genetic origin was suspected. The panel successfully established a genetic diagnosis for 52 patients (34%) but could not identify causal or probable variants for the remainder. The authors attribute this relatively moderate diagnostic yield to the characteristically high genetic and phenotypic heterogeneity of hearing loss (many causal variants could occur outside coding regions or on genes not included on the panel), and note promising opportunities for future development, both in terms of refining panel design and in shedding further light on the different inheritance patterns associated with the phenotype. The results provide compelling support for the use of DNA sequencing gene panels in newborn screening for hearing loss.

The articles published in this Research Topic have applied genomics technologies to study various diseases and medical applications and demonstrate the effective use of genomics in diverse fields of research. There is a need to create such publishing platforms for emerging researchers, where they can promote their ideas and showcase their research capabilities to a global audience. This topic paves the way for future endeavours in this direction, encouraging young researchers to experience the technicalities of paper writing, submission, and the editorial process, as well as the art of effective science communication.

